# Navigating the life stage after stroke: From Life 2.0 to stroke prevention models of care — A qualitative exploration of younger and middle-aged adult stroke patients' experiences and recommendations

**DOI:** 10.1016/j.qrmh.2025.100017

**Published:** 2025-07-29

**Authors:** Sarah Ibrahim, Danielle D’Amico, Lindsey Zhang, Syeda Hashmi, Angela Verven, Sharon Ng, Troy Francis, Aleksandra Stanimirovic, Jasper R. Senff, Sanjula Singh, Jonathan Rosand, Leanne K. Casaubon, Keithan Sivakumar, Valeria Rac, Aleksandra Pikula

**Affiliations:** a Program for Health System and Technology Evaluation, Toronto General Hospital Research Institute, Toronto (TGHRI), ON, Canada; b Research Institute of the McGill University Health Centre (RI-MUHC), Montreal, Quebec, Canada; c Institute of Health Policy, Management and Evaluation (IHPME), Dalla Lana School of Public Health, University of Toronto, Canada; d Centre for Advancing Collaborative Healthcare & Education (CACHE), University of Toronto, Toronto, ON, Canada; e Rotman Research Institute, Kimel Family Centre for Brain Health & Wellness, Baycrest Academy for Research and Education, Toronto, ON, Canada; f Faculty of Medicine, University of Ottawa, Ottawa, ON, Canada; g Faculty of Medicine, University of Toronto, Ontario, Canada; h Jay and Sari Sonshine Centre for Stroke Prevention & Cerebrovascular Brain Health, Toronto Western Hospital, University Health Network, Canada; i Harvard Chan School of Public Health, Boston, MA, USA; j Henry and Allison McCance Center for Brain Health, Massachusetts General Hospital, Boston, MA, USA; k Department of Neurology, Massachusetts General Hospital, Boston, MA, USA; l Broad Institute of MIT and Harvard, Cambridge, MA, USA; m Center for Genomic Medicine, Massachusetts General Hospital, Boston, MA, USA; n Department of Neurology and Neurosurgery, Brain Center Rudolf Magnus, University Medical Center Utrecht, Utrecht, NL, the Netherlands; o Department of Medicine, Division of Neurology, University of Toronto, Toronto, Ontario, Canada; p Department of Neurology, Toronto Western Hospital, Toronto, ON, Canada; q Krembil Brain Institute, University Health Network, Toronto, Ontario, Canada

**Keywords:** Younger and middle-aged adults, Stroke, Qualitative research, Experience

## Abstract

**Background:**

Global stroke incidence has been rising among adults 65 years of age or younger. A dearth of research exists exploring and understanding younger and middle-aged adults’ lifestyle-related knowledge and habits along with associated facilitators and/or barriers with the adoption, maintenance, and support needs for development of new brain health interventions, which this study sought to address.

**Methods:**

A qualitative study was conducted, followed by virtual, semi-structured focus groups. Data collection and analysis were performed using Goffman's dramaturgical theory to guide the inductive thematic data analysis.

**Results:**

A total of 12 participants comprised the sample. Four themes emerged: 1) *Front stage: Life 2.0*, 2) *Back stage: Unseen and invisible challenges*, 3) *Scripts and audience reaction: Dualism of social influence*; and 4) *Setting: Standard of care, but to who’s standard?*

**Conclusion:**

Findings contributed to a deeper understanding of factors influencing the adoption of healthy habits and approaches to reconceptualize and re-design brain health interventions that meet the needs, preferences, and priorities of this population.

## Introduction

Stroke is traditionally perceived of as a disease of older adulthood (Bukhari et al., 2023). However, globally, the fastest-growing stroke burden is among adults 65 years of age and younger (Feigin et al., 2022). Notably, stroke results in detrimental physical, mental, cognitive, and psychosocial health consequences (Sasikumar & Pikula, 2018) that affect younger and middle-aged adults and, even more so, age-related life tasks (e.g., school, relationships, parenting, and driving) during the most productive and dynamic period of their lives and for much longer than older adults (Amoah et al., 2023). This parallels undesirable implications of stroke and associated complications on healthcare systems, work productivity, and economies worldwide (Khan et al., 2021).

Globally, an estimated 90 % of the stroke burden is attributed to modifiable risk factors (MRFs) (e.g., hypertension, obesity, smoking, poor diet, and low physical activity) (Pikula et al., 2024) which are lifestyle associated, making them preventable and potentially addressed through Lifestyle Medicine (LM). LM is an evidence-based clinical discipline that applies medical, environmental, motivational, and behavioral principles to comprehensively support the adoption of healthy lifestyle habits and address the underlying causes of MRFs for diseases and conditions such as stroke (Adsul et al., 2020). Adherence to healthy lifestyle practices, habits, and lifestyle-related interventions have been associated with the reduction of stroke risk and reoccurrence (Pikula et al., 2024).

The importance of behavior change, and the long-term adoption of healthy lifestyle habits have been emphasized by a myriad of evidence-based clinical guidelines (e.g., the American Heart Association [Lloyd-Jones et al., 2022] and the Heart and Stroke Foundation of Canada [Heart and Stroke Foundation of Canada, 2021]). However, there is a paucity of research that explores 1) lifestyle-related knowledge and behaviors to support primary and/or secondary stroke prevention among younger and middle-aged adults with prior stroke or at higher stroke risk and 2) recommendations for post-stroke and brain recovery and prevention interventions and models of care based on their experiences.

Emerging literature on younger and middle-aged adult stroke patients’ lifestyle knowledge and habits is not promising (Tack et al., 2025). In our recent cross-sectional study (N = 104, 52 % women, mean age 47.5 %, and 53.8 % with stroke) exploring younger and middle-aged adult stroke patients’ lifestyle-related knowledge and behaviors, we observed variability in LM-related knowledge (with poor knowledge of nutrition and physical activity recommendations) and the adoption of healthy behaviors with gender-, age-, and stroke status-related differences (Ibrahim, Sivakumar et al., 2024). Additionally, in another cross-sectional study (N = 101, 50 % ≤65 years of age, two-thirds overweight or obese) only 50 % of the participants correctly identified stroke risk factors (Althomali et al., 2024). Further, the majority of participants did not believe maintaining a healthy weight or engaging in physical activity was important, while of those who smoked, nearly half did not feel smoking cessation was important to reduce stroke risk (Althomali et al., 2024).

Although the majority of studies mentioned above collected data on lifestyle-related knowledge and behaviors through quantitative approaches such as surveys, qualitative approaches are imperative to attain valuable in-depth insight and understanding and to further explore people’s thoughts, perspectives, and experiences that cannot be explained or captured using quantitative research approaches (Renjith et al., 2021). Thus, the aim of this qualitative study was to address the current gap in research and to 1) explore younger and middle-aged adult stroke patients’ current lifestyle-related knowledge and lifestyle habits, 2) examine facilitators and barriers regarding adaptation of healthy lifestyle habits, and 3) support needs for stroke and brain-related interventions and models of care, based on participants’ experiences.

## Methods

### Study design

We conducted a qualitative study with semi-structured focus groups as part of a larger mixed-methods study that explored younger and middle-aged adults’ lifestyle-related knowledge and behaviors along with the facilitators and barriers that may influence the adoption and/or maintenance of such habits. We followed the “Standards for Reporting Qualitative Research” checklist (O’Brien et al., 2014) to ensure the reporting was comprehensive and transparent.

### Theoretical framework

The theoretical framework informing our data analysis was Erving Goffman’s dramaturgical theory (Goffman, 1990) which offers a unique perspective on social interaction by framing daily life as a kind of theatrical performance. Goffman writes that people actively shape and present different roles and identities while adopting varying scripts based on the social situation and audience, akin to actors performing on a stage. Key elements of the theory include front and back stage, scripts, roles, and audience. “Front stage” refers to the public persona—performing and adhering to social norms and expectations. “Back stage” is where people express their true selves privately and away from the pressures and scrutiny of the audience. “Scripts” are predefined patterns of behaviors influenced by cultural norms, protocols, and personal experiences. “Role” and “audience” refer to the different functional positions persons occupy depending on the context, setting, and audience.

### Sample and setting

The study population consisted of adults who were 1) of working age (65 years or younger), 2) had a hemorrhagic or ischemic stroke more than 90 days prior to recruitment or were at higher stroke risk (e.g. carried diagnosis of transient ischemic attack [TIA], vascular malformations, or unruptured aneurysm), 3) were being seen at either the stroke prevention clinic or neurovascular clinic at one of the largest stroke centers in Ontario, Canada; and 4) able to communicate in English. Participants were excluded from the study if they had advanced cognitive impairment (defined as mRS [Modified Rankin Scale] > 4 and/or a diagnosis of dementia) that would limit the provision of informed consent and/or had a brain injury (e.g., subarachnoid hemorrhage) due to trauma.

### Participant recruitment

Participants who took part in the quantitative phase of the initial mixed-methods study (an online survey) (N = 104) were asked if they were interested in taking part in a focus group to further explore their perspectives and understanding of brain health, healthy lifestyle habits, associated facilitators and barriers, recommendations for designing post-stroke and brain health-related interventions, and models of care pertaining to gender differences. A total of 23 participants from the quantitative phase of the initial study expressed an interest in taking part in the focus groups. The coordinator contacted all potential participants by telephone and/or email based on the contact information provided to further explain the details of the qualitative study, inquire about their interest, and answer any questions and/or concerns. Eleven people did not respond following three attempts of contact by the coordinator.

### Ethical considerations

The study was approved by the institutional Quality Improvement Review Committee (QIRC#23–0518). Verbal consent was obtained from all participants to take part in the focus group and to be recorded for data collection purposes.

### Data collection and analysis

**Fig. 1:**
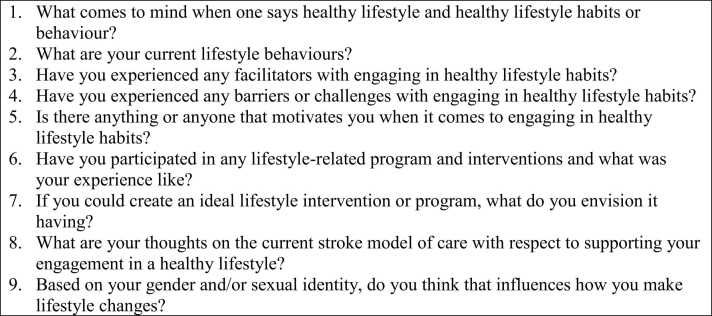
Sample of semi-structured interview questions.

Data were collected through virtual focus groups via Microsoft Teams to reduce travel costs and to allow participants to take part from different geographic areas of Toronto, Ontario. The interviewer did not have any contact with participants prior to this study. Participants with mild aphasia were included, and aphasia-friendly communication techniques were employed using close-captioning via Microsoft Teams and screen sharing, highlighting questions being asked. The focus groups were 60 to 75 min in duration. A semi-structured interview guide was used (Fig. 1), guided by findings from the quantitative phase of the initial study (Ibrahim, Sivakumar et al., 2024). The focus groups were audio-recorded and transcribed verbatim. Once the audio-recordings were transcribed, they were permanently deleted to maintain participants’ confidentiality and anonymity. Participants were offered a $10 gift card as a token of appreciation for their time and participation.

Concurrent data collection and analysis were employed to accommodate the emergence of codes and themes from the data and to the point of data saturation (Ravindran, 2019). Identifiable information (e.g., participants’ names and name of providers and institutions) were removed from the transcripts, and a participant identification number was assigned for each participant. An inductive thematic analysis was conducted using the six phases outlined by Braun and Clarke (2006): 1) familiarization with the data, 2) generation of initial codes, 3) search for themes, 4) review of themes, 5) definition and naming of themes, and 6) production of the report. The transcripts were independently reviewed and coded by the first author and second author. The first two authors met on a regular basis to discuss the codes and patterns and to organize them into themes, continually revisiting the data to ensure that themes were well supported by testimony among the focus groups. Any discrepancies were resolved through consensus. The authors’ backgrounds are in nursing, education, interventions, and qualitative and mixed methods research.

Validity and reliability were maintained through our application of credibility, dependability, conformability, and transferability. By “credibility,” we mean peer-debriefing and reflexivity through journaling; this allowed the researchers to reflect on their respective backgrounds, feelings, perspectives, and potential biases throughout the data collection and analysis process (Anney, 2014). For “dependability,” we established code-to-code procedures, i.e., initial data coding, waiting two weeks to re-code the same data, and then comparing both sets of codes (Anney, 2014). “Confirmability” consisted of an audit trail and reflexivity through journaling. For “transferability,” we used thick description which is a strategy that allows for clear explanation of the research process (e.g., data collection and analysis procedures) by the team (Anney, 2014).

### Participant characteristics

A total of four focus groups (with three or four participants per group) were conducted with a final sample size of 12 participants. Eight participants identified as men, with an average age of 45 years (range 33–59). Six participants were married, and seven had no child responsibility. Eight participants identified as White and being Canadian born with English as their first language. Ten participants had experienced a stroke (Table 1).

**Table 1:** Demographic and Clinical Characteristics.

	N = 12
**Gender**	
Women	4 (33.3 %)
Men	8 (66.7 %)
**Age**	
Mean (SD)	45.17(9.94)
Range	33 −59
**Marital Status**	
Missing	2
Single	4(40 %)
Married/Cohabitating	6(60 %)
**Level of Education**	
Missing	2
Completed college/university	7(70 %)
Post-graduate Degree	3(30 %)
**Child Responsibility**	
Missing	2
No responsibility	7(70 %)
Moderate responsibility	1(10 %)
Very responsible	1(10 %)
Full responsibility	1(10 %)
**Canadian or Foreign Born**	
Missing	2
Canadian-born	8(80 %)
Foreign-born	2(20 %)
**English Language**	
Missing	2
English first language	8(80 %)
English second language	2(20 %)
**Ethnicity**	
Missing	2
White	8(80 %)
East Asian	1(10 %)
Latin American	1(10 %)
**Employment Status**	
Missing	2
Manual paid work	1(10 %)
Non-manual paid work	2(20 %)
Unemployed, looking for work	2(20 %)
On leave of absence	3(30 %)
Retired	1(10 %)
On disability	1(10 %)
**Diagnosed with a Stroke**	
No	2(16.7 %)
Yes	10(83.3 %)

## Results

**Fig. 2:**
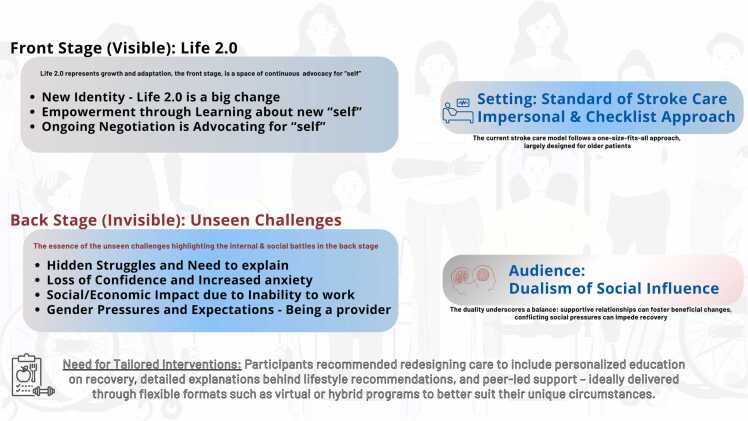
Overview of themes and subthemes.

Four themes emerged, guided by Goffman’s dramaturgical theory (Fig. 2). These themes align with the core elements of the theory—front stage, backstage, scripts, audience, and setting. Collectively, they frame how participants perform, manage, and negotiate their new self-identities, experiences, and recommendations post-stroke.

### Front stage: Life 2.0

Participants spoke of their post-stroke identity as "Life 2.0"--a term they used to capture the transformation and reconfiguration of their new self identity. Having a new self-identity and sense of purpose, shaped by both internal adaptations and external social expectations, was a facilitator to support participants’ recovery. They spoke of working through their post-stroke mental health challenges, loss of confidence, exploring how much and how far they can push themselves safely, and learning to live with their new post-stroke identity. Most participants also discussed how their new self-identity was vastly different from their identity pre-stroke in terms of abilities, pace, capacity, and work—all of which influenced their personas and roles in their front stage. This shift reveals the ongoing negotiation of their self-identity in social spaces, coupled with the new script they are forced to devise, learn, and perform post-stroke, as described by one participant:

People talk about being their 2.0 person—their life 2.0 now that they had a stroke. There’s a mental challenge to deal with what that new person looks like and for some of us who are goal oriented. I’m struggling with how much more is possible, and I know there’s no answer. What's the new you look like and how hard you keep pushing to try and pick up ground. (Participant 2)

While participants optimistically discussed their “Life 2.0,” they also described the emotional labor and frustration with having to navigate through their post-stroke personal and professional social circles (i.e., their audience) in a socially acceptable manner, using their front stage persona. Emotional labor and frustration stemmed from the invisibility of their stroke-related impairments (e.g., memory loss and changes in concentration), general lack of awareness among the audience, and the concentrated efforts behind their performance. The disconnect between appearance and reality—aligning with Goffman’s notion of impression management—and the constant monitoring and adjustment of behavior to maintain a desired image and persona was expressed by two participants:

I think the barrier is making people around me understand that I'm different. Having to kind of constantly remind [them] that I have a brain injury. That kind of constant feeling like being an outsider and reminding people that I'm different [pause] can be really, really exhausting (Participant 3).

They [other people] only kind of see the tip of the iceberg and don't see how you're keeping it all together underneath, how you're working very hard to present yourself and they forgot. They just forget that we're working really hard to seem normal. (Participant 2)

The metaphor “tip of the iceberg” speaks to the enormous challenge participants encounter in maintaining front-stage face. Participants’ front stage has the potential to collapse under the weight of the emotional labor, fatigue, frustration, and cognitive overload. For instance, participants found themselves having to constantly prompt others to slow down when communicating, reluctantly disclosing to friends and others (e.g., government personnel regarding disability-related matters) of their stroke in order to follow conversations, interact with others, and to obtain pertinent medical, government, and work-related information. The act of “coming out,” though perceived to be necessary among participants to maintain the front stage and preserve their sense of belonging, was perceived to be stigmatizing and intrusive and, in turn, a barrier to their post-stroke recovery and well-being, as a participant explained:

If you visually look like they don't have a disability, that in itself is a detriment. I don't want to visually look like I have a disability, but I use the term “coming out” multiple times. I'll be carrying groceries out as I can't do this, and I ask my wife for help because that's my dead hand. I mean, it's not dead. I can do things with it and she was like I forgot. Well, she forgot because I'm able to do things, but certain things are just too much. My own spouse is forgetting. I don't want to remind my friends, I don't want to [pause] I'm not going to walk around with a sign on my neck, and I don't think anyone else wants to walk around with a sign on their necks saying there's an issue…” (Participant 1)

### Back stage: Unseen and invisible challenges

This theme centered around the many unseen and invisible challenges occurring backstage that young and middle-aged adult participants experienced post-stroke and kept hidden from their front-stage persona. The challenges centered around confidence, social life, the inability to work, and gender expectations. Most participants described losing self-confidence during their post-stroke recovery, and this was amplified by the audience and context as two participants expressed:

I lost a lot of confidence. My profession is, like, very high stress before all this happened. And now, I'm much more conscious and much more careful to quickly spot stress and anxiety rising and using tactics to step away. I just don't have the capacity for the stress that I did before. You know, you’re going at 150 miles, and now, I literally can't do it. I just can't operate that way any more, so I've dialed back workloads, stress, anxiety through other strategies to rebuild the foundations or the workflows that I had before so that they're, like, more conducive to keeping stress manageable. (Participant 5)

In particular, participants who identified as men described how backstage, they emotionally struggled with their inability to return to work post-stroke. This left them without the financial resources to participate in social activities such as dating and attending with friends, for example:

I had my stroke on my first day of my vacation from work and have been on disability since. I can barely survive on that, and I think that affects me mentally, too. Because nature stops you from a lot of things in life. [pause] You still need money to get to places. You got to pay for transportation to go somewhere. You got to pay for a drink, food, you know, meeting up with your friends. Going for lunch costs money. Meeting someone, you know, to date even, you still need money to take them out, and that's one part. But I kind of stopped doing that, looking for somebody or even meeting somebody because I don't have the financial funds to do any of that, and it sometimes feels that I'm alone, too. (Participant 7)

As demonstrated in this quote, backstage experiences contributed to participants’ feeling of loneliness, loss of hope in finding a life partner, and isolation. They also had a negative impact on participants' mental health and ability to maintain lifestyle habits (e.g., social connectedness and stress management).

Dramaturgical theory helps us to understand how the backstage thus becomes a place of both refuge and struggle where participants grapple with the dissonance of their internal reality and external expectations. This was captured by another participant:

I had to stop working at age 38. Well, like, almost 17 years ago now. That's a long time, and that's young to have to stop working. [pause] It was really hard because I was really young. And without sounding horribly sterotypical, as a male, it's even harder. We're in a society that when a male isn't working…. It's changing thankfully, but [when] a male isn't working, it's looked upon differently than a female. And that's wrong, but I'm very sorry. It’s still reality in in a lot of ways…. (Participant 1).

### Scripts and audience reaction: Dualism of social influence

The focus of this theme was the dualism (both positive and negative) of social influence on participants’ lifestyle-related behaviors to support their recovery. Following dramaturgical theory, social scripts guide the performance through the delineation of roles amidst different contexts, with the reaction of the audience reinforcing or challenging the performance. As such, participants described how their family and friends were role models, providing scripts for facilitating healthy lifestyle habits and behaviors. As one person stated:

A lot of good models in my family. I'm lucky. My wife, my parents, her parents, my brothers. Everyone. And so, I look at my family, especially the older ones who are just crushing it at that age. I'm just, like, man, I just want to be like that. So that's motivating. I have good role models and things like that. And my wife who's around my age. And we connected and fell in love with all these activities that we did together. And I see you're doing it. And I'm, like man, I just want to keep doing that with her. (Participant 7)

Here, supportive audiences reinforced the front and back stages of participants’ recovery. Notably, this translated into emulating and embodying their family’s healthy habits as their own (e.g., grandparents living long and healthy lives) and using this as a “motivator,” which posed as a facilitator to their recovery.

Healthy habits (e.g., decreasing the consumption of processed food and engaging in physical activity) are aligned with the six pillars of LM: nutrition, physical activity, stress management, sleep health, substance use, and social connectedness. Modeling lifestyle habits were thus facilitators in participants’ post-stroke recovery and secondary stroke prevention. Some participants also indicated that working with therapists (e.g., neuropsychologists) aided in their psychological and emotional post-stroke journey.

Conversely, some participants expressed how their lifestyle habits and behaviors were negatively influenced and disrupted by their social audiences—both personal and professional. Such negative influence was more prevalent when participants were unable to effectively control their environment such as travelling for work and conferences:

When I'm traveling and I'm at conferences and around a bunch of people, and we're going out and ordering like 20 apps [appetizers] and drinking, although that's usually my fault. Like, it's really easy to just consume a day’s worth of calories in five minutes just because everybody else is reaching for it and you're distracted and talking. So, I find that's probably the most impactful, negatively impactful aspect. (Participant 9)

Unvoiced in this quotation is that younger and middle-aged adults do not want to be excluded from social interactions and activities, yet, at times, such engagements were quite disruptive to the maintenance of their healthy eating habits. Participants’ feelings of not wanting to be excluded aligns with the increasing evidence of daily social interactions being important mechanisms and influences on physical health, including the frequency and severity of physical symptoms (Berkman et al., 2000, Zhaoyang et al., 2019).

This perspective was similarly reported by another participant who stated that, “Work friends. I know especially like they want to go out…. I'm always forced to go and, you know, eat something that I don't really want to or just like, you know, disrupting” (Participant 3). Here, we an see the dual role of the audience on shaping participants’ front stage performance: Supportive audiences validate and sustain participants’ recovery scripts, whereas unsupportive or unaware audiences unintentionally undermine them.

### Setting: Standard of care, but to whose standard?

In discussing their experiences, participants extensively highlighted the challenges and frustrations with the current standard of stroke care and what constitutes as "standard" of care. They expressed that the existing stroke care model, which is the setting and, in turn, the backdrop to participants’ performance(s), was impersonal and revolved around checklists, standard practices, and adherence to standard procedures by healthcare providers (HCPs). The impositions from the setting and the standardized script clashed with participants’ needs. This was expressed by two participants:

They don't know you from anybody. They're just giving the typical party line: “Well, you're going to need to eat this or not eat this. You need to lead a healthier lifestyle.” And as I hear the words, I almost cringe because I think to myself, “You don't even know me.” (Participant 11)

I've had a couple of strokes [pause] and it was sort of a well, “Don't smoke.” Well, I don't smoke. “Well, don't drink a lot.” Well, I drink like one drink a week? “Uh, get some exercise.” Is five days a week enough? [The HCPs] go through a sort of a checklist, and it was like “no” for all those. It was like they had to read the speech. And when they didn't get the answers they wanted, you know, I'm not overweight or wasn't overweight at the time either and obviously very active, and they don’t get the answers, they are, like, “Oh, we don't know, I did my thing, I said what I had to.” (Participant 2)

Many participants also noted a lack of education on psychosocial and emotional aspects post-stroke, which affected the backstage experience. This was coupled with the existing language predominantly geared towards older adults and their roles and responsibilities stemming from a script that assumes that stroke occurs among older adults and that the roles, responsibilities, interests, commitments, and goals (e.g., returning to work) of young and middle-aged adults are the same as those of older adults. This led to feelings of demoralization, discouragement, confusion, frustration, and uncertainty of the best next steps to support their recovery. Consequently, participants found themselves having to advocate for their own health (even while still in the acute phase of their recovery), independently search the internet for relevant information and organizations, and seek alternative and non-acute care resources to support healthier lifestyle habits, return to work, and affect overall post-stroke recovery:

When I was being discharged, I didn't really get much of a discharge meeting when they let me out of the hospital. The doctor said, “Can you walk? Do you feel OK?” And that was pretty much it. [pause] I didn't really get a lot of information, and they didn't really explain a lot in terms of what I could and couldn't do. They just basically said, “Don't raise your heart rate too much. ”And I was, like, “OK, what does that mean? I don't know.” (Participant 7)

I think in terms of explaining and caring for me, where it really kind of fails is the preventative aspect of it. I don't think it [healthcare system] ever assumes that anyone under the age of, like, 50 is at risk of a stroke because I think in the public knowledge, we know that cholesterol, high cholesterol is bad. But I don't think anyone ever says you could actually have a stroke at the age of, like, 30 to 40 because of it. And even my family physician, I don't think they ever used that as a risk. They were just saying, “Hey, we need to mitigate this,” but they never explained why. (Participant 12)

In these quotes, we can see how the healthcare setting, as the stage, constrained participants’ front and backstage performances as well as their recovery scripts. The variation in the institutional script and participants’ experiences created a dissonance where participants had to navigate the rigid setting, advocate for themselves, and seek alternative supports. As such, participants recommended redesigning and innovating post-stroke interventions to support the adoption of positive and healthy lifestyle behaviors tailored to their unique needs, preferences, and goals.

Participants emphasized need for a focus on education and on the psychosocial and emotional aspects of post-stroke recovery to alleviate fears of stroke recurrence, depression, anxiety, isolation, panic attacks, emotional distress, loneliness, frustration, and anger. Additionally, they highlighted the importance of providing more information in support of HCP recommendations (e.g., activities to do or avoid) to better understand the rationale, which might, in turn, support more long-term sustainment of lifestyle habits. Inclusion of such information would support backstage experiences of participants, allowing them to engage in honest conversations about their experiences, new self-identity, and front-stage performance and persona:

….information like, “Here are some things that people have been through and the mental health issues they face. These are a couple of things that you can expect to feel, like feeling fear and lack confidence—common things that have been observed. You're having fear and anxiety and you're like, ‘Is this just me, or is did everyone feel like this?’” I would feel better if I heard from more people and this is totally normal and happens to other people. (Participant 9)

Participants also highlighted the importance of peer-led interventions to offer participants a sense of hope, belonging, and connection that cannot be provided by HCPs, family members, or peers who have not experienced a stroke, thus supporting an authentic backstage performance and environment:

…being around other people who've been through stroke experiences is something I'm interested in, and I get a lot out of it. No one can really understand what it's like to have gone through this, and it's nice to be around such people. (Participant 11)

Peer-led interventions would thus allow for impression management that is supportive of exploring a new self-identity in a non-judgemental and safe environment. Such interventions would also afford participants opportunities to learn strategies to effectively manage and cope with anxiety (based on symptoms and post-stroke sequelae), access resources and tools from individuals with similar experiences, and combat stigma. This in turn, would support the rehearsal of new roles that participants can bring to their front-stage personas, interactions, and performances.

Virtual and/or hybrid modes of delivering these interventions were also perceived to be beneficial for minimizing the time commitment for participants, increasing convenience, and allowing for a better balance of personal and professional responsibilities. These modes and interventions were seen to support flexible role performance as well as shared backstage spaces where participants can 1) engage in their recovery without disrupting other aspects of their social identity, 2) process their experiences among persons with similar experiences, and 3) rehearse new coping strategies. These recommendations were expressed by several participants, for example:

Health anxiety in general. Just like always wondering, could it happen again? Or I was having really bad anxiety about my memory. And I was, like, is that because of the aneurysm? Am I losing my memory? Am I losing my long-term? and I was really panicked about that. I think it was just lack of sleep that was really affecting my cognitive abilities for a while and which is getting better now. But, yeah, I think talking that through with people would help and maybe calm that anxiety a little bit. (Participant 3)

I participated in a group in [name of company] with persons with lived experience with ADHD, and we had meetings where people would share their experiences and the tools that help them…. And I felt were valuable. (Participant 7)

## Discussion

This study revealed a deeper understanding of the complex ways young and middle-aged adults navigate their post-stroke experiences, identities, facilitators, barriers, and support needs for stroke-related interventions and the current model of care. Stroke recovery depends on well-coordinated care. Aided by a dramaturgical perspective, integrated stroke care can align back stage processes such as funding and team collaboration with front stage interactions, ensuring patients experience seamless support relating to their individual needs and preferences. Closing gaps between provider efforts and patient perceptions has the potential to improve rehabilitation and outcomes and sets the stage for better communication and stroke prevention interventions.

### Facilitators to positive and healthy lifestyle behaviors and post-stroke recovery

Facilitators in support of positive and healthy lifestyle behaviors and participant’s post-stroke recovery in this study were influenced by development of new self-identities combined with social connectedness. Psychological ownership is rarely considered in disease state or chronic illness (Karnilowicz, 2011). Psychological ownership, however, is as salient as the disease process, and recovery often requires the patient to gain a sense of control and assume the role of new ownership, which can be challenging, yet rewarding, and provide a new and productive outlook on life.

The new post-stroke self-identities found in this study among stroke patients is consistent with other qualitative systematic reviews (Kuluski et al., 2014, Lou et al., 2017, Ibrahim et al., 2024). Interestingly, social connectedness also posed as a facilitator in this study. Participants expressed the positive influence their family had on behavioral changes—encouraging and motivating them throughout their post-stroke journey to not just live longer, but healthier. The findings from this study are also consistent with a qualitative descriptive study in Uganda with participants between 20–40 years wherein adoption of healthy lifestyle habits among young and middle-aged adults were related to social support from family (e.g., cooking, dietary changes) and the community (e.g., formation of physical activity groups) (Ndejjo et al., 2022).

### Barriers to positive and healthy lifestyle behaviors and post-stroke recovery

Barriers to the adoption of healthy lifestyle habits and overall post-stroke recovery for young and middle-aged adult stroke participants in this study centered on the 1) negative social influence and societal expectations (for men, in particular) and 2) current standards of stroke care. While also a facilitator, social influence was found to be a barrier for the study participants’ adoption of healthy and positive lifestyle behaviors, especially with their eating habits. This finding is again consistent with Ndejjo et al. (2022) in which lack of role models and peers and culture-related factors (e.g., exercise not being commonly acceptable, particularly for women) negatively influenced participants’ adoption of healthy and positive lifestyle behaviors. This was further coupled with unhealthy food choices offered in communal gatherings and the consumption of alcohol among peers (Ndejjo et al., 2022), emphasizing the critical role that diverse sociocultural influences have on the adoption of healthy and/or unhealthy practices.

As noted above, gender expectations influence post-stroke role perceptions among participants. Specific societal pressures that some of the men in this study articulated were related to finances, work, and the perception of masculinity. Such findings are consistent with a literature analysis of Canadian men’s chronic disease self-management strategies (Zanchetta et al., 2017). Of note, men’s sense of masculinity, self-image, and perceived fulfillment of expected social roles were threatened by living with a chronic disease, highlighting the complex gender and health-specific dynamics (Zanchetta et al., 2017) pertaining to the adoption of healthy lifestyle behaviors and support of post-stroke recovery.

Specific to the current design of post-stroke interventions and model of care, participants indicated the vital need to shift away from the current standard of care that is permeated with pro forma checklists and processes that are often not perceived among patients as being conducive to the adoption of healthy lifestyle habits or in support of post-stroke recovery. Furthermore, participants expressed an interest in having more age-specific and tailored resources and information that was geared towards them as young and middle-aged adults, their life situation (e.g., careers, families), and unique challenges and needs (e.g., psychosocial and emotional, desire to return to work, and dating).

Participants also recommended peer-led post-stroke initiatives and interventions. Such a shift in the current design of post-stroke interventions and model of care would support the much needed and overdue paradigm shift of value-based health care which places the patient and their health at the epicenter of health care (Esposti & Banfi, 2020). Understanding the needs of patients and strategically optimizing related interventions, initiatives, and models of care have been linked to improving quality of life, health outcomes, patient satisfaction, and reduction of healthcare costs (Teisberg et al., 2020). These findings are consistent with other phenomenological qualitative research study that aimed to explore the unmet needs of young adults(N = 10; 21–27 years; 1–15 years post-stroke) (Amoah et al., 2023). In particular, availability of age-specific information, resources, and initiatives were reported as gaps in post-stroke recovery.

Furthermore, several participants reported utilizing social networks with young and middle-aged adults living with various health conditions which afforded them a sense of belonging, reduced their isolation and boredom, and improved their overall social connectedness (Amoah et al., 2023). These findings are consistent with a cross-sectional study (Keating et al., 2021) and a qualitative study (Shipley et al., 2020) demonstrating that unmet needs of young and middle-aged adult stroke patients centered on limited gender- and age-specific information and care as well as peer-support, which was a widely acceptable and preferential intervention approach.

## Implications

**Fig. 3:**
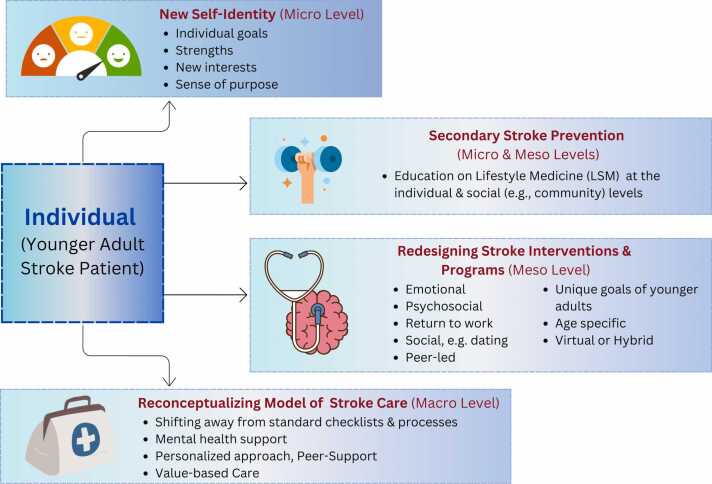
Potential implications at the micro, meso and macro level.

Our findings have several social innovations-related implications (Fig. 3). First, interventions and initiatives should focus on supporting young and middle-aged adult stroke patients’ new self-identity post stroke, with emphasis on their abilities, strengths, areas of previous and new interests, and overall purpose in all phases and roles of their life. Such interventions can be developed through a co-design approach, which is increasingly being integrated in development of stroke-related interventions (Singh et al., 2024).

Second, it is important for further integration of LM interventions in primary and secondary stroke prevention to continue to enhance lifestyle-related knowledge and to support the adoption of positive and healthy lifestyle behaviours, all the while accounting for facilitators of and barriers to behavioral change. Lifestyle medicine education and behavioral interventions can also be extended to various social settings such as community health centers, work places, recreational centers, and faith-based centers (see also, Tomlinson, 2022) appreciating the dual social influence (both positive and negative) on young and middle-aged adult stroke patients’ adoption of healthy behaviors.

Third is the overdue need to reimagine and design current stroke interventions that are geared towards young and middle-aged adults—their phase of life, responsibilities, aspirations, and needs—which can be accomplished through a co-design and participatory approach (Singh et al., 2024).

Fourth, it is crucial for stroke interventions and initiatives to focus on the identified unmet needs of young and middle-aged adults such as emotional and psychosocial factors that are age-specific (e.g., dating, work and family responsibilities and ability to return to work) in order to address barriers that preclude the adoption of healthy behaviors and hinder post-stroke recovery.

Fifth, peer-led interventions may be a beneficial approach to post-stroke recovery. Peer-support interventions have been extensively used to improve health outcomes for people living with chronic diseases (Werfalli et al., 2020, Wijekoon et al., 2020). Peer-led initiatives involve oversight by providers and people with lived experience who deliver the intervention or program and through this, promote interpersonal connections by mutual experiences, goals, and needs, facilitating social connectedness with current patients (May et al., 2023).

Finally, interventions should prioritize shifting away from the current model of care and towards one that is more value-based, personalized, and tailored. This can be accomplished through different means such as integrating patient reported health outcomes (PROMs) (Dawson et al., 2010) and patient-reported experience measures (PREMs) (Cornelis et al., 2021) in clinical practice and as part of assessment to guide practice and care. This can also be achieved by re-evaluating the current standards of care, processes, and practices that do not account properly for the stroke demographic changes and that are primarily geared towards older adults.

## Limitations

There are several limitations to this study. First, the findings are based on results of a qualitative study conducted in a single academic health sciences center in Ontario, Canada. Second, the experiences of the participants in this study may differ from those who did not agree to take part in the research study. Finally, the majority of participants identified as White, with English being their first language. These limitations highlight the need for further recruitment efforts to ensure recruitment of diverse participants.

## CRediT authorship contribution statement

**Troy Francis:** Writing – review & editing, Writing – original draft. **Aleksandra Stanimirovic:** Writing – review & editing, Writing – original draft, Conceptualization. **Jasper R. Senff:** Writing – review & editing, Writing – original draft. **Sanjula Singh:** Writing – review & editing, Writing – original draft. **Jonathan Rosand:** Writing – review & editing, Writing – original draft. **Leanne K. Casaubon:** Writing – review & editing, Writing – original draft. **Sarah Ibrahim:** Writing – review & editing, Writing – original draft, Methodology, Formal analysis, Conceptualization. **Keithan Sivakumar:** Writing – review & editing, Writing – original draft, Conceptualization. **Danielle D’Amico:** Writing – review & editing, Formal analysis. **Valeria Rac:** Writing – review & editing, Writing – original draft, Methodology, Conceptualization. **Lindsey Zhang:** Writing – review & editing, Visualization, Data curation. **Aleksandra Pikula:** Writing – review & editing, Writing – original draft, Investigation, Funding acquisition, Conceptualization. **Syeda Hashmi:** Writing – review & editing, Writing – original draft, Conceptualization. **Angela Verven:** Writing – review & editing, Writing – original draft, Visualization. **Sharon Ng:** Writing – review & editing, Writing – original draft, Data curation.

## Funding

This work was supported by funding from the University of Toronto Division of Neurology (The Slamen-Fast New Initiatives in Neurology).

## Declaration of Competing Interests

The authors declare that they have no known competing financial interests or personal relationships that could have appeared to influence the work reported in this paper.
